# Application of Radiomics in Prognosing Lung Cancer Treated with Epidermal Growth Factor Receptor Tyrosine Kinase Inhibitors: A Systematic Review and Meta-Analysis

**DOI:** 10.3390/cancers15143542

**Published:** 2023-07-08

**Authors:** Ting-Wei Wang, Ming-Sheng Hsu, Yi-Hui Lin, Hwa-Yen Chiu, Heng-Sheng Chao, Chien-Yi Liao, Chia-Feng Lu, Yu-Te Wu, Jing-Wen Huang, Yuh-Min Chen

**Affiliations:** 1 School of Medicine, National Yang Ming Chiao Tung University, Taipei 112, Taiwan; eltonwang1@gmail.com (T.-W.W.); sam090904sam@gmail.com (M.-S.H.); chiuhwayen@gmail.com (H.-Y.C.); hschao2@vghtpe.gov.tw (H.-S.C.); 2 Institute of Biophotonics, National Yang Ming Chiao Tung University, Taipei 112, Taiwan; ytwu@nycu.edu.tw; 3 Department of Radiation Oncology, Taichung Veterans General Hospital, Taichung 407, Taiwan; irene1992yi@gmail.com; 4 Department of Chest Medicine, Taipei Veterans General Hospital, Taipei 112, Taiwan; 5 Institute of Biomedical Informatics, National Yang Ming Chiao Tung University, Taipei 112, Taiwan; 6 Department of Biomedical Imaging and Radiological Sciences, National Yang Ming Chiao Tung University, Taipei 112, Taiwan; wl03151783@gmail.com (C.-Y.L.); alvin4016@ym.edu.tw (C.-F.L.)

**Keywords:** non-small cell lung cancer, radiomics, EGFR tyrosine kinase inhibitors, treatment outcome, computed tomography

## Abstract

**Simple Summary:**

Lung cancer is one of the most common cancers and can be difficult to treat. One of the treatment methods uses drugs that target a protein called the epidermal growth factor receptor, but the results vary from patient to patient. In this research, we used a technique called radiomics, which involves analyzing detailed scans of the patients’ tumors, to see if we can predict who will respond best to these drugs. We reviewed previous studies and found that this method was promising, with patients showing certain patterns on their scans more likely to have longer periods without disease progression. However, more research is needed to confirm these findings and develop reliable methods of using these scans in clinical practice.

**Abstract:**

In the context of non-small cell lung cancer (NSCLC) patients treated with EGFR tyrosine kinase inhibitors (TKIs), this research evaluated the prognostic value of CT-based radiomics. A comprehensive systematic review and meta-analysis of studies up to April 2023, which included 3111 patients, was conducted. We utilized the Quality in Prognosis Studies (QUIPS) tool and radiomics quality scoring (RQS) system to assess the quality of the included studies. Our analysis revealed a pooled hazard ratio for progression-free survival of 2.80 (95% confidence interval: 1.87–4.19), suggesting that patients with certain radiomics features had a significantly higher risk of disease progression. Additionally, we calculated the pooled Harrell’s concordance index and area under the curve (AUC) values of 0.71 and 0.73, respectively, indicating good predictive performance of radiomics. Despite these promising results, further studies with consistent and robust protocols are needed to confirm the prognostic role of radiomics in NSCLC.

## 1. Introduction

### 1.1. Overview of Lung Cancer and Its Global Burden

Lung cancer, characterized by uncontrolled growth of abnormal cells in the lungs, remains one of the leading causes of cancer-related deaths worldwide [1]. The two major types of lung cancer are non-small cell lung cancer (NSCLC), accounting for approximately 85% of cases, and small cell lung cancer (SCLC), accounting for the remaining 15% [2]. According to the World Health Organization, lung cancer is responsible for over 1.7 million deaths annually, making it a significant public health concern [3]. Factors contributing to lung cancer incidence include tobacco smoking, exposure to radon gas, asbestos, and air pollution, as well as genetic predisposition [4]. Despite advances in early detection and treatment strategies, the overall five-year survival rate of patients with lung cancer remains relatively low [5]. This highlights the need for improved diagnostic and prognostic tools, such as radiomics, to better predict treatment outcomes and guide personalized therapy decisions for patients receiving targeted therapies such as tyrosine kinase inhibitors (TKIs) [6].

### 1.2. Role of Tyrosine Kinase Inhibitors (TKIs) in Lung Cancer Treatment

Tyrosine kinase inhibitors (TKIs) have emerged as effective targeted therapies for lung cancer, particularly for patients with non-small cell lung cancer (NSCLC) harboring specific genetic mutations [7]. These small-molecule drugs inhibit the activity of tyrosine kinases, a group of enzymes responsible for the activation of several cellular processes, including cell growth, proliferation, and survival [8]. In lung cancer, certain genetic mutations, including those in the epidermal growth factor receptor (EGFR), lead to overactivation of tyrosine kinases, promoting tumor progression and malignancy [9].

EGFR plays a crucial role in the regulation of cell proliferation, differentiation, and survival. Mutations or overexpression of EGFR have been identified in several types of cancers, including a significant proportion of NSCLCs. These mutations lead to the constitutive activation of the EGFR pathway, driving cell proliferation and inhibiting apoptosis, thus promoting tumorigenesis [10,11]. Simultaneously, the PI3K/AKT/mTOR pathway, another critical signal transduction system, is frequently dysregulated in many types of cancers, including NSCLC. This pathway, when activated, promotes cell growth, proliferation, survival, and metabolism. Aberrations in this pathway, often occurring due to mutations in the PIK3CA gene or loss of the tumor suppressor PTEN, can lead to overactivation of the PI3K/AKT/mTOR pathway, promoting tumorigenesis [12,13].

TKIs, including gefitinib, erlotinib, afatinib, and osimertinib, have been approved for the treatment of NSCLC [14]. These drugs target these overactive kinases, thereby blocking the signals that contribute to tumor growth, and are particularly effective in patients with EGFR mutations, ALK rearrangements, and ROS1 rearrangements [15]. By targeting the specific molecular drivers of lung cancer, TKIs offer a personalized and potentially less toxic alternative to traditional chemotherapy [16].

However, not all patients respond equally to TKI therapy, and some may eventually develop resistance [17]. This challenge underlines the importance of exploring other targets like the PI3K/AKT/mTOR pathway and the application of combinatorial treatments. As a result, there is growing interest in identifying prognostic factors, such as radiomic features, that can help predict treatment outcomes and optimize therapeutic strategies for lung cancer patients treated with TKIs [18].

### 1.3. Importance of Radiomics in Predicting Treatment Outcomes

Radiomics is a rapidly evolving field that focuses on the extraction and analysis of quantitative imaging features from medical images, such as computed tomography (CT), magnetic resonance imaging (MRI), and positron emission tomography (PET) scans [19]. These features, often referred to as radiomic signatures, can provide valuable insights into the underlying biology, heterogeneity, and treatment response of tumors [18]. By harnessing the power of advanced computational algorithms and machine learning techniques, radiomics has the potential to significantly improve the accuracy and precision of cancer prognosis and predict treatment outcomes [6].

In the context of lung cancer treated with TKIs, radiomics offers several advantages for predicting patient outcomes. First, it allows for non-invasive assessment of tumor characteristics, which may help identify patients who are more likely to respond to TKI therapy [20]. Second, radiomic signatures may serve as early biomarkers of treatment response, enabling clinicians to monitor therapeutic efficacy and adjust treatment plans accordingly [21]. Finally, the integration of radiomic features with clinical and molecular data can lead to the development of comprehensive prognostic models; that can help guide personalized treatment decisions for patients with lung cancer [22].

As the field of radiomics continues to grow, its potential to improve prognostic accuracy and precision in lung cancer patients treated with TKIs is becoming increasingly evident [23]. Ongoing research and advancements in this area hold promise for enhancing the overall management of lung cancer and optimizing the use of targeted therapies such as TKIs [24].

### 1.4. Objectives of the Meta-Analysis

Recently, a surge in studies has focused on the potential clinical implications of radiomic features extracted from computed tomography (CT) images in patients with non-small cell lung cancer (NSCLC). These investigations emphasize the correlation between radiomic features and the response or outcomes following EGFR-TKI targeted therapy interventions. The primary objective of this research was to conduct a comprehensive systematic review of the current landscape of radiomic studies, particularly in the context of predicting the response or outcomes for NSCLC patients receiving targeted therapy. This review encompasses an evaluation of the quality of radiomic studies, utilizing the Quality in Prognosis Studies (QUIPS) tool for image mining research and the radiomics quality scoring (RQS) tool as benchmarks. Moreover, quantitative analysis was performed to evaluate the effectiveness of radiomics in forecasting the response and outcomes of targeted therapy in this cohort of patients.

## 2. Materials and Methods

### 2.1. Search Strategy and Selection Criteria

#### 2.1.1. Databases and Search Terms

This systematic review and meta-analysis were conducted in accordance with the PRISMA guidelines [25]. The study has not been registered. To ensure meticulous adherence to these guidelines, checklists corresponding to PRISMA were utilized and are presented in Supplemental Table S1. To identify relevant studies for this meta-analysis, a comprehensive literature search was conducted using the following electronic databases: PubMed, Embase, Web of Science, and the Cochrane Library. The search strategy included a combination of keywords and Medical Subject Headings (MeSH) terms related to lung cancer, tyrosine kinase inhibitors, radiomics, and prognosis. The search terms included lung cancer, non-small cell lung cancer, NSCLC, Tyrosine kinase inhibitors, TKIs, Gefitinib, Erlotinib, Afatinib, Osimertinib, Radiomics, radiomic features, radiomic signature, texture analysis, prognosis, survival, treatment outcome, response, and prediction. The search was conducted from the inception of each database (i.e., the earliest record) to the date of the database search (4 April 2023). The search strategy was adapted for each database, and additional studies were identified by searching the references of the included articles and relevant reviews. The detailed search strategy for this systematic review and meta-analysis is provided in the Supplemental Table S2.

#### 2.1.2. Inclusion and Exclusion Criteria

Studies were included in the meta-analysis if they met the following criteria: (a) study design: original research articles, including retrospective and prospective studies, (b) population: adult patients (≥18 years old) with NSCLC treated with EGFR-TKIs, (c) intervention: radiomics analysis using CT scans, and (d) outcome measures: association between radiomic features and treatment outcomes, including treatment response and progression-free survival (PFS). The exclusion criteria were as follows: (a) studies not published in English, (b) review articles, conference abstracts, case reports, editorials, and supplementary materials, (c) insufficient data or lack of relevant outcome measures, and (d) studies with overlapping or duplicate patient populations.

Two independent reviewers (T. W. and M. H.) screened the titles and abstracts, followed by a full-text assessment to determine eligibility for inclusion. Any discrepancies between the reviewers were resolved through discussion or consultation with a third reviewer, if necessary.

### 2.2. Data Extraction and Quality Assessment

#### 2.2.1. Data Extraction Process

For each included study, relevant data were extracted by two independent reviewers using a standardized data extraction form. The extracted information included the following: (a) study characteristics: authors, year of publication, study duration, study country, study design, and sample size, (b) patient demographics: age, sex, stage of lung cancer, (c) EGFR-TKI therapy details: type of EGFR-TKI used, (d) radiomic features: imaging modality, software, segmentations, feature extraction methods, and radiomic signatures, (e) clinical and molecular data: smoking history and other relevant clinical factors, and (f) outcome measures: progression-free survival. Any discrepancies in data extraction were resolved through discussion or consultation with a third reviewer, if necessary.

#### 2.2.2. Quality Assessment

The methodological quality of the included studies was appraised using the Quality in Prognosis Studies (QUIPS) tool, which assesses the risk of bias and applicability concerns in prognostic studies [26], as well as the radiomics quality score (RQS), a radiomics-specific quality assessment tool [6]. The QUIPS tool encompasses six domains: study participation, study attrition, prognostic factor measurement, outcome measurement, study confounding, and statistical analysis and reporting. Each domain was examined for the risk of bias, while applicability concerns were addressed for the first three domains. The 16-component RQS tool evaluates the validity and potential bias of radiomics studies. Each study was assigned a score for each RQS component, and scores were then summed to obtain a total score. Two independent reviewers (T. W. and M. H.) undertook the quality assessment, and any discrepancies were resolved through discussion or, if necessary, consultation with a third reviewer.

### 2.3. Meta-Analysis

Three meta-analyses were performed with the included studies: (1) a meta-analysis of studies investigating the use of radiomics to compare PFS of target therapy between high- and low-risk groups in the validation datasets, which was measured by the pooled hazard ratio (HR) and a 95% confidence interval (CI) using the random-effects model; and (2) a meta-analysis of predictive performance of radiomics models using the area under curve (AUC) for median progression-free survival and Harrell’s Concordance Index (c-index) for progression-free survival using a fixed effects model. The AUC is derived from the receiver operating characteristic (ROC) curve, which is a graphical representation of a model’s true positive rate (sensitivity) against its false positive rate (1-specificity) at various threshold settings. The AUC quantifies the overall performance of a model with a value of 1 indicating perfect classification; a value of 0.5 suggests no better performance than random chance. Harrell’s concordance index is a measure of the performance of biomarkers or models in which the outcome is a time-to-event measure that includes censored data [27]. Multiple c-indices or the AUC were reported for a given dataset (e.g., due to multiple machine learning methods being utilized for model development within the same manuscript); the best-performing model that included radiomics features within the model was chosen for inclusion in the meta-analysis. The hazard ratio was transformed to logarithms scale, while the c-index and AUC were treated as the expected values, and the 95% CI was used to back-calculate the SD with the corresponding T-score from a Student’s T Distribution with n-1 degrees of freedom. If a single study did not provide a 95% CI or SD, a standard error of the mean (SE) was reported instead. The SD was calculated by multiplying the square root of the sample size by the SE.

### 2.4. Statistical Analysis

Heterogeneity between studies was assessed using Cochran’s Q test and quantified using the I^2^ statistic. The I^2^ statistic measures the percentage of variability in effect estimates attributable to heterogeneity rather than sampling error. I^2^ values of 25%, 50%, and 75% were indicative of low, moderate, and high heterogeneity, respectively. If significant heterogeneity was detected (I^2^ > 50%), a random-effects model (DerSimonian–Laird method) was used employed for the meta-analysis. Otherwise, a fixed-effects model was used (Mantel–Haenszel method). Combined effects were calculated, and a two-sided *p* value < 0.05 was considered to indicate statistical significance [28]. Publication bias was assessed only if there were more than 10 studies, as more than 10 studies were required to detect funnel plot asymmetry [29]. All analyses were performed using Review Manager (RevMan) [Computer program], version 5.4, Cochrane Collaboration; 2020.

## 3. Results

### 3.1. Study Selection and Characteristics

#### 3.1.1. Flow Diagram of Study Selection

**Figure 1:**
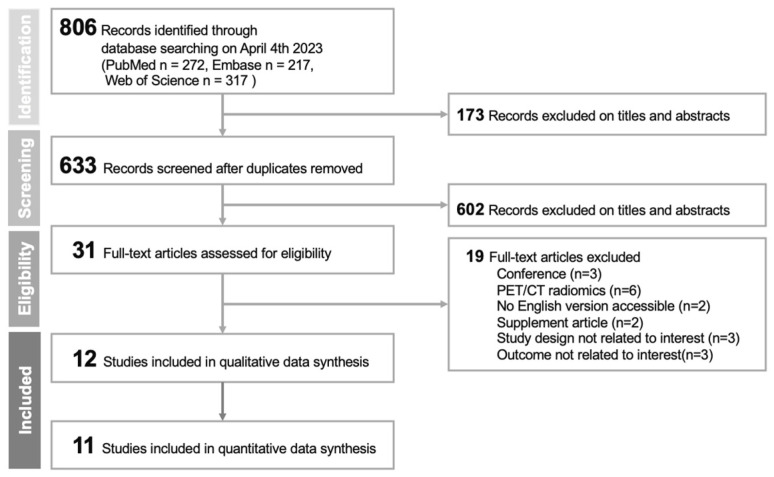
PRISMA 2020 flowchart of included studies.

Figure 1 presents the flow diagram outlining the study selection process for this systematic review and meta-analysis. The initial search across databases and additional sources yielded 806 articles. After removing duplicates, 633 articles were retained for further assessment. Titles and abstracts of these articles were screened, resulting in the exclusion of 602 articles. Subsequently, 31 full-text articles were thoroughly examined for eligibility, with 19 articles being excluded for reasons detailed in Figure 1. Ultimately, 12 studies were included in this systematic review and meta-analysis [30,31,32,33,34,35,36,37,38,39,40,41].

#### 3.1.2. Characteristics of Included Studies

The 12 studies enrolled a total of 3111 patients with advanced NSCLC treated with EGFR-TKI [30,31,32,33,34,35,36,37,38,39,40,41]. The basic characteristics of the studies are summarized in Table 1 and Table 2. All of the 12 studies were retrospective. The median patient age ranged from 55 to 67.5 years, and the proportion of patients who were female ranged from 51.2% to 64%. The majority of patients had adenocarcinoma. CT was performed before beginning EGFR-TKI treatment. All of the studies used progression-free survival as the endpoint, and the median PFS ranged from 8.1 to 13.1 month.

#### 3.1.3. Radiomics and Image Analysis

The radiomic and image analysis details of the included studies are presented in Table 2. In terms of the region of interest selection, tumor segmentation was performed in ten studies [30,31,32,33,35,36,37,39,40,41], while two studies did not include tumor segmentation [34,38]. For the studies with segmented tumors, primary tumors alone were segmented in five studies [30,31,33,39,41], while the others defined regions of interest [32,35,36,37,40]. Clinical features were incorporated into six studies [30,32,36,37,39,41]. Traditional handcrafted radiomics methods were used in six studies [30,32,35,37,39,40], and temporal changes in radiomics were accounted for in two studies [31,41]. Deep learning models were employed for the extraction of deep learning radiomics in four studies [33,34,36,38]. External validation of the models was conducted in only four studies [31,33,38,39]. Conventional statistical methods were utilized for radiomic analysis in all studies, except for three studies that employed deep learning [30,33,36] and machine learning techniques [31].

### 3.2. Quality Assessment Results

**Figure 2:**
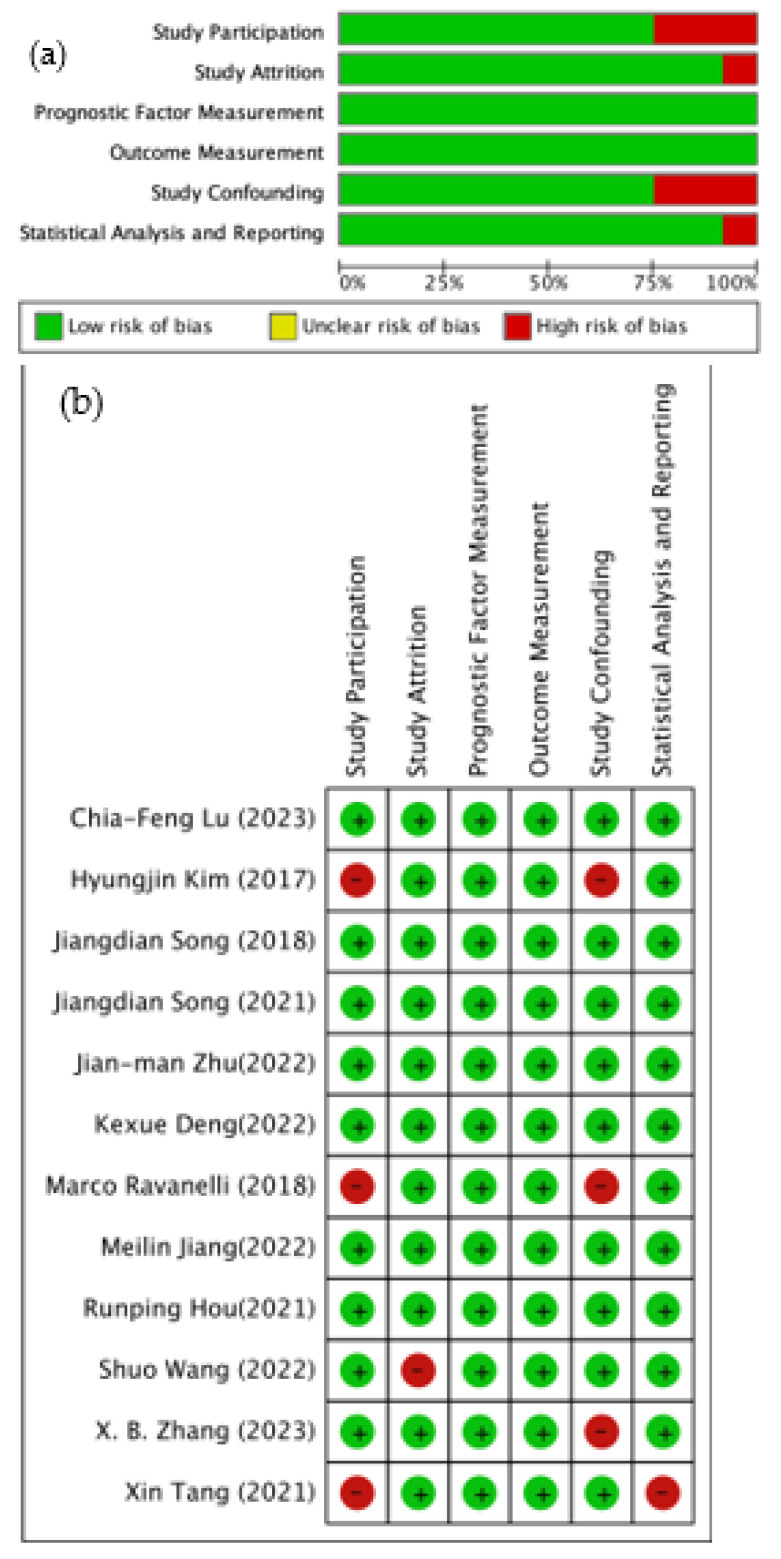
Quality assessment. (**a**) Risk of bias for individual studies. (**b**) Summary of risk of biases [30,31,32,33,34,35,36,37,38,39,40,41].

Quality in Prognosis Studies (QUIPS) assessment of the 12 studies is shown in Figure 2 [30,31,32,33,34,35,36,37,38,39,40,41]. The assessment of individual studies showed that there were low risk bias and fair application concerns for most of the assessed criteria, except for higher risk of study participants and confounding measurement in three studies. Statistical analysis and reporting also showed high risk of bias in one study (Figure 2a). A summary of the risk of bias for all studies is shown in Figure 2b.

Table 3 presents the individual and total RQS scores for all included studies as evaluated by two reviewers. The mean score for the 12 studies was 11.67 (range: 9–15). A majority of the studies provided well-documented image acquisition protocols. Baseline scans were used for image acquisition in all studies, with two studies [31,41] incorporating additional imaging at follow-up time points. Manual segmentation was employed in nine studies (75%), and semi-automatic segmentation was used in one study (8.3%), while two studies (12.6%) did not use segmentation methods. Feature dimension reduction or adjustment was conducted in all studies, and for deep learning studies, it was assumed that the models inherently possessed feature selection characteristics. Clinical features were integrated into the radiomic models in six studies (50%) [30,32,36,37,39,41], and five of these studies suggested that the combination of clinical data and radiomic features could enhance the models’ predictive performance. The clinical characteristics included age, gender, smoking, histology, Eastern Cooperative Oncology Group (ECOG) performance status, M staging, N staging, EGFR mutation, clinical staging, and blood tests. The correlation between tumor biology and radiomic features was investigated and discussed in one study (8.3%). Most studies carried out cutoff analysis to stratify patients into low- and high-risk groups. Regarding model assessment, discrimination statistics were frequently provided, while calibration statistics were less frequently mentioned. Validation of radiomics signatures was conducted in 12 studies (100%), with 4 studies (25%) utilizing external datasets from other institutions. Nine studies demonstrated clinical utility, and three performed cost-effectiveness analyses. In terms of open science and data availability, three of the included studies made their code open source, contributing to the transparency and reproducibility of their findings [33,38,39]. The utilization of open-source approaches in these studies, which includes providing access to code, models, and some data, markedly contributes to the transparency and reproducibility of the research. In the study by Kexue D et al. [33], an EfficientNetV2-based Survival Benefit Prediction System (ESBP) was developed. They provided a comprehensive set of resources including code for training, validation, and testing, tools for CT image reading and processing, and a subset of their anonymized dataset. This allows for reproducibility of their results and potential application or adaptation of their methodology by other researchers, bolstering the reusability of their model. On the other hand, the study by Jiangdian S in 2021 [38] focused on providing the code for training their deep learning model, although the weights of the trained model or data were not explicitly provided. In addition, the study also shared code for extracting the radiomic features used in their analysis. Although the final model was not directly provided, with the provided code and similar data, researchers can reproduce their methodology and calculate the desired outcomes. In another instance, one study surpassed standard practices by openly sharing both the calculated features and representative regions of interest (ROIs) [39]. This significantly enhances the reusability of their models and allows for a deeper and more comprehensive analysis by other researchers.

### 3.3. Radiomic Features and Prognostic Performance

**Figure 3:**
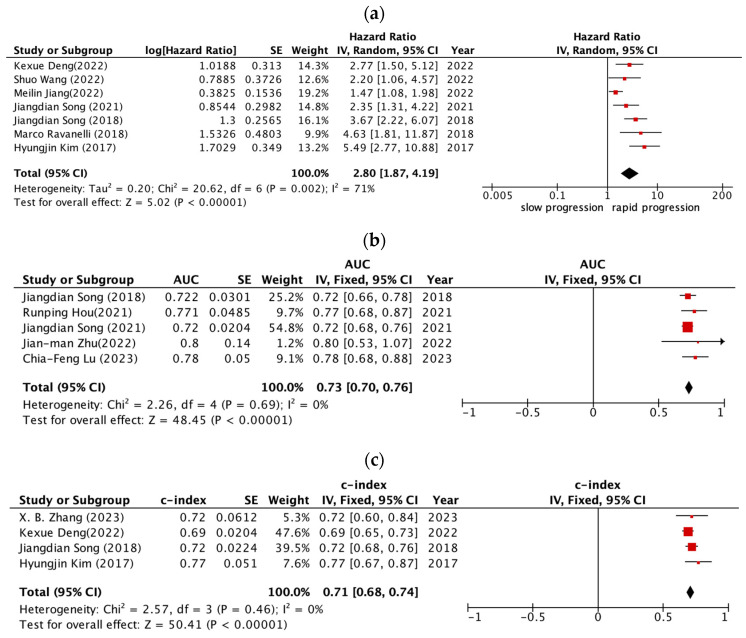
Forest plots of the predictive performance of radiomics in progression-free survival of NSCLC patients treated with target therapy. (**a**) Hazard ratio (**b**) median PFS (**c**) c-index [32,33,34,35,36,37,38,40,41,42,43].

The patients could be stratified into low- and high-risk groups by radiomic models. The first meta-analysis of comparing the target therapy outcome between the two groups showed that the pooled HR was 2.80 (95% CI 1.87–4.19, *p* < 0.001) for PFS (seven studies; Figure 3a). The I^2^ statistic implied moderate heterogeneity among the studies (I^2^ = 71%, *p* = 0.002). The second and third meta-analysis, which evaluated the predictive power of prognosis model with the AUC and c-index, showed the pooled AUC was 0.73 (95% CI 0.70–0.76, *p* < 0.001) for median PFS and the c-index was 0.71 (95% CI 0.68–0.74, *p* < 0.001) (four studies; Figure 3c). The I^2^ statistic implied low heterogeneity among the studies (I^2^ = 0%, *p* = 0.69; I^2^ = 0%, *p* = 0.46 for the AUC and c-index).

## 4. Discussion

### 4.1. Quality of Radiomic Studies: QUIPS and RQS Evaluation

In our systematic review, we assessed the quality of the included radiomic studies using the Quality in Prognosis Studies (QUIPS) tool and the radiomics quality score (RQS). These evaluations provided insights into the methodological rigor and reproducibility of the studies, which are essential for establishing the reliability and generalizability of the findings. Overall, the mean RQS of the included studies was 11.67 (range: 9–15), indicating a moderate level of quality. Most studies have reported well-documented image acquisition protocols, and the majority have employed segmentation methods, such as manual or semi-automatic approaches. Feature dimension reduction or adjustment was performed in all studies, with deep learning models assumed to possess inherently feature selection characteristics. Additionally, half of the studies integrated clinical features into radiomic models, which suggested that combining clinical data with radiomic features could improve predictive performance. However, there were some limitations to the quality assessment. Notably, only four studies employed external datasets for validation, and only a few studies discussed the correlation between tumor biology and radiomic features or demonstrated the clinical utility and none addressed cost-effectiveness of the radiomic models. The limited availability of open science and data sharing is also concerning.

### 4.2. Summary of Main Findings of Meta-Analysis

This meta-analysis focused on assessing the role of radiomics in predicting treatment outcomes and response in NSCLC patients receiving targeted therapies, specifically EGFR-TKIs. Our results demonstrated that radiomic models can effectively stratify patients into low- and high-risk groups. The high-risk group was associated with poorer progression-free survival (PFS) than the low-risk group. Furthermore, quantitative evaluation of the predictive performance of radiomic models, as measured by the area under the curve (AUC) and concordance index (c-index), was generally satisfactory. The pooled hazard ratio (HR) for PFS between the low- and high-risk groups was 2.40 (95% CI 1.89–2.80, *p* < 0.001), indicating that the high-risk group had a significantly worse PFS outcome. The I^2^ statistic revealed high heterogeneity among studies (I^2^ = 71%). The pooled AUC for the radiomic models in predicting median PFS was 0.73 (95% CI 0.70–0.76, *p* < 0.001), and the pooled c-index was 0.71 (95% CI 0.68–0.74, *p* < 0.001). These values suggest that the radiomic models exhibited a good predictive performance. The I^2^ statistic for both the AUC and c-index showed low heterogeneity among the studies (I^2^ = 0%). Overall, these findings highlight the potential utility of radiomic models in identifying high-risk patients with receiving EGFR-TKIs, which could contribute to personalized treatment strategies and improve patient outcomes.

### 4.3. Clinical Implications

To the best of our knowledge, this is the first systematic review and meta-analysis to focus on the application of radiomics in the prognosis of NSCLC treated with targeted therapy. The findings of our study have several noteworthy implications for the management of patients with NSCLC receiving targeted therapies. The ability to stratify patients based on their risk profiles, as demonstrated by radiomic models, allows healthcare professionals to make more informed treatment decisions. This approach can potentially facilitate personalized treatment plans that consider each patient’s unique risk factors and tumor characteristics [42,43]. Moreover, the integration of radiomic signatures with clinical and molecular data can contribute to the development of comprehensive prognostic models [21,44]. These models can provide a more accurate prediction of treatment response and outcomes, thereby guiding the optimization of therapeutic strategies for NSCLC patients treated with EGFR-TKIs [45,46]. It is important to note that external validation and standardized protocols are essential to ensure the reliability and reproducibility of radiomic models [19]. Future studies should emphasize the need for standardized image acquisition, preprocessing, and feature extraction methods, as well as rigorous validation strategies to improve the clinical utility of radiomic models in the management of NSCLC [47,48].

### 4.4. Future Directions and Study Limitations

To further advance the field of radiomics in predicting treatment outcomes for NSCLC patients receiving targeted therapies, future studies should address the limitations identified in this review. These include increasing the use of external validation datasets, exploring the biological relevance of radiomic features, and enhancing data-sharing practices. Moreover, the incorporation of emerging technologies, such as artificial intelligence and machine learning algorithms, can potentially improve the accuracy and predictive power of radiomic models. The quantitative outcomes from this systematic review and meta-analysis highlight the potential of radiomic models in predicting treatment outcomes in NSCLC patients receiving targeted therapies. For instance, the pooled hazard ratio (HR) for PFS was 2.80, with a 95% confidence interval (CI) of 1.87–4.19 (*p* < 0.001). Furthermore, the pooled AUC and c-index values were 0.73 (95% CI: 0.70–0.76, *p* < 0.001) and 0.71 (95% CI: 0.68–0.74, *p* < 0.001), respectively, suggesting satisfactory predictive performance of the radiomic models.

In addition to the current focus on tyrosine kinase inhibitors, it is equally pertinent to consider the potential role of other EGFR inhibitors such as monoclonal antibodies (mAbs) like cetuximab and panitumumab in NSCLC treatment. These mAbs have shown promise in clinical settings, and it would be insightful to examine their clinical implications through radiomic analysis. Future research should endeavor to integrate these therapies into radiomic predictive models, which could potentially widen the scope of personalized treatment strategies for NSCLC patients. Furthermore, these future investigations could also shed light on the comparative efficacy and therapeutic value of TKIs versus mAbs, enhancing our understanding and management of NSCLC.

Despite our comprehensive analysis, there are some limitations to consider. First, the number of studies included was relatively small, which may have limited the generalizability of our findings. Additionally, the limited number of studies may have contributed to potential country bias, where certain regions are overrepresented while others are underrepresented. Second, we observed high heterogeneity in some meta-analyses, which could be due to differences in study populations, imaging protocols, or radiomic methodologies. Finally, the inclusion of studies published only in English may have introduced language bias and potentially excluded relevant findings from non-English publications.

## 5. Conclusions

In summary, our systematic review and meta-analysis demonstrated the potential of radiomics in predicting treatment outcomes and responses in patients with NSCLC receiving targeted therapies such as EGFR-TKIs. Radiomic models effectively stratify patients into low and high-risk groups, providing a valuable tool for guiding personalized treatment decisions. However, the quality of the included studies varied, with some limitations identified in terms of external validation, biological relevance, and data-sharing practices. To further advance the field of radiomics and optimize its utility in clinical practice, future studies should address these limitations and focus on integrating emerging technologies, such as artificial intelligence and machine learning algorithms. Moreover, large-scale, multicenter studies with diverse populations would help validate and generalize radiomic models, ensuring their applicability across various clinical settings. Ultimately, the continued development and refinement of radiomic models hold the promise of improving the overall management of NSCLC patients and optimizing the use of targeted therapies such as EGFR-TKIs. By harnessing the predictive power of radiomics, clinicians can better tailor treatment strategies to individual patients, potentially enhancing treatment efficacy and patient long-term outcomes in the long run.

## Figures and Tables

**Table 1:** Basic characteristics of studies included in the systematic review and meta-analysis.

Author	Dataset	Study Duration	Country	Study Design	Patients	Age (Years)	Female (%)	Smoker (%)	Stage	Adeno (%)	EGFR-TKI	Median PFS (Years)
Chia-Feng L (2023) [30]	D	2018~2019	Taiwan	Retrospective	270	67.5	158 (59)	69 (26)	IIIB~IV	263 (97.4)	First line First, second Gen	11.5
X. B. Z (2023) [31]	D	2015~2020	China	Retrospective	131	NR	74 (57)	33 (25)	II~IV	131 (100)	First, second, third Gen	11.1
	E	2015~2020	China	Retrospective	41	NR	24 (59)	9 (22)	II~IV	41 (100)	First, second, third Gen	13.1
Jian-man Z (2022) [32]	D	2016~2019	China	Retrospective	100	NR	64 (64)	23 (23)	IIIB~IV	100 (100)	First line EGFR TKI	10
Kexue D (2022) [33]	D	2010~2021	China	Retrospective	478	58	286 (60)	112 (23)	IV	451 (94)	First, second, third Gen	NA
	E	2010~2021	China	Retrospective	92	60	52 (57)	22 (24)	IV	86 (93)	First, second, third Gen	NA
Shuo W (2022) [34]	D	2009~2018	China	Retrospective	600	59	349 (58.2)	150 (25)	I~IV	574 (95.7)	First line First Gen	11.42
Meilin J (2022) [35]	D	2013~2018	China	Retrospective	187	55	107 (57.2)	57 (30.5)	III~IV	187 (100)	First Generation	12
	V	2018~2019	China	Retrospective	38	57	23 (60.5)	12 (31.6)	III~IV	38 (100)	First Generation	11.8
Runping H (2021) [36]	D	2013~2017	China	Retrospective	239	61	142 (59.4)	55 (23)	IIIA~ IVB	239 (100)	First line EGFR TKI	9
	V	2013~2017	China	Retrospective	100	61	68 (68)	17 (17)	IIIA~ IVB	100 (100)	First line EGFR TKI	9
Xin T (2021) [37]	D	2017~2021	China	Retrospective	273	57	167 (61.2)	55 (20.1)	IV	NA	Osimertinib	13.3
Jiangdian S (2021) [38]	D	2010~2017	China	Retrospective	145	NA	87 (60)	60 (41)	IV	135 (93)	First, second, third Gen	9.9
	E	2010~2017	China	Retrospective	101	NA	60 (59)	21 (21)	IV	99 (98)	First, second, third Gen	9.2
	E	2010~2017	China	Retrospective	96	NA	55 (57)	17 (18)	IV	92 (96)	First, second, third Gen	8.2
Jiangdian S (2018) [39]	D	NA	China	Retrospective	117	NA	73 (62)	53 (45)	IV	NA	EGFR-TKI	8.1
	E	NA	China	Retrospective	101	NA	60 (59)	21 (21)	IV	NA	EGFR-TKI	9.2
	E	NA	China	Retrospective	96	NA	55 (57)	17 (17)	IV	NA	EGFR-TKI	8.2
Marco R (2018) [40]	D	2008~2016	Italy	Retrospective	55	66	29 (58)	23 (46)	IV	55 (100)	First line EGFR TKI	10.5
Hyungjin K (2017) [41]	D	2005~2015	Korean	Retrospective	48	61	25 (51.2)	22 (45.8)	NA	NA	First line EGFR TKI	9.7

D, development dataset; V, validation dataset; E, external validation dataset; NA, not applicable; Adeno, adenocarcinoma; EGFR-TKI, epidermal growth factor receptor tyrosine kinase inhibitor; Gen, generation; PFS, progression-free survival.

**Table 2:** Summary of details of radiomic and image analyses.

Author	Segmentation	VOI	Clinical Feature	Software	Radiomics	Validation	Classifier	Endpoints
Chia-Feng L (2023) [30]	Manual	Primary tumor	N, M, histology, TP, MCV	Multimodal Radiomics Platform	Radiomics	Split sample	DeepSurv	PFS
X. B. Z (2023) [31]	Semi-automatically	Primary tumor	None	Syngo.via Frontier, Radiomics, version 1.2.5, Siemens Healthineers	Delta Radiomics	External validation	Random survival forest	PFS
Jian-man Z (2022) [32]	Manual	ROI	age, sex, stage, smoking, mutations, TKI, outcome	Pyradiomics	Radiomics	Cross validation	logistic regression model	PFS
Kexue D (2022) [33]	Manual	Primary tumor	None	EfficientNetV2 architecture (deep learning)	Deep learning Radiomics	External validation	EfficientNetV2 architecture	PFS
Shuo W (2022) [34]	NA	Whole lung	None	FAIS (deep learning)	Deep learning Radiomics	Split sample	LASSO-Cox	PFS
Meilin J (2022) [35]	Manual	ROI	None	Pyradiomics	Radiomics	Split sample	Cox-proportional hazard	PFS
Runping H (2021) [36]	Manual	ROI	age, sex, smoking, clinical stages, molecular status	3D CNN (deep learning)	Deep learning Radiomics	Split sample	3D CNN	PFS
Xin T (2021) [37]	Manual	ROI	PS and M	NA	Radiomics	Cross validation	stepwise regression	PFS
Jiangdian S (2021) [38]	NA	Whole slice	None	BigBiGAN	Deep learning Radiomics	External validation	LASSO-Cox	PFS
Jiangdian S (2018) [39]	Manual	Primary tumor	smoke, N	programmed algorithms	Radiomics	External validation	LASSO-Cox	PFS
Marco R (2018) [40]	Manual	ROI	None	TexRAD	Radiomics	Cross validation	Cox-proportional hazard	PFS
Hyungjin K (2017) [41]	Manual	Primary tumor	age, baseline tumor diameter, and treatment response	Medical Imaging Solution for Segmentation and Texture Analysis	Delta Radiomics	Cross validation	Cox-proportional hazard	PFS

NA, not applicable; VOI, volume of interest; N, N staging; M, M staging; TP, total protein; MCV, mean corpuscular volume; TKI, tyrosine kinase inhibitor; PS, performance status; LASSO-Cox, Least Absolute Shrinkage and Selection Operator- Cox proportional hazards, CNN, convolution neural network; PFS, progression-free survival.

**Table 3:** Details of radiomic quality score.

	Domain 1				Domain 2		Domain 3				Domain 4			Domain 5		Domain 6	
Author	Image Protocol Quality	Multiple Segmentation	Phantom Study on All Scanner	Imaging at Multiple Time Points	Feature Reduction or Adjustment for Multiple Testing	Validation	Multivariable Analysis with Non Radiomics Features	Detect and Discuss Biological Correlates	Comparison to ‘Gold Standard’	Potential Clinical utility	Cut-off Analyses	Discrimination Statistics	Calibration Statistics	Prospective Study Registered in a Trial Database	Cost-Effectiveness Analysis	Open Science and Data	Total
Chia-Feng L (2023) [30]	0	0	0	0	3	2	1	0	0	1	1	2	0	0	0	0	10
X. B. Z (2023) [31]	1	1	0	1	3	3	1	0	0	1	1	2	0	0	0	0	14
Jian-man Z (2022) [32]	1	1	0	0	3	2	0	0	0	0	1	2	0	0	0	0	10
Kexue D (2022) [33]	0	1	0	0	3	3	1	0	0	1	1	2	0	0	0	2	14
Shuo W (2022) [34]	1	0	0	0	3	3	0	1	0	1	0	0	0	0	0	0	9
Meilin J (2022) [35]	1	0	0	0	3	2	1	0	0	1	1	2	0	0	0	0	11
Runping H (2021) [36]	1	0	0	0	3	2	1	0	0	1	0	2	0	0	0	0	10
Xin T (2021) [37]	1	1	0	1	3	2	1	0	0	1	0	2	1	0	0	0	13
Jiangdian S (2021) [38]	1	0	0	0	3	3	1	0	0	1	1	2	1	0	0	1	14
Jiangdian S (2018) [39]	0	1	0	0	3	4	1	0	0	1	1	2	1	0	0	1	15
Marco R (2018) [40]	1	0	0	1	3	2	1	0	0	0	1	2	0	0	0	0	11
Hyungjin K (2017) [41]	1	0	0	1	3	2	1	0	0	0	1	2	0	0	0	0	11

## Supplementary Materials

The following supporting information can be downloaded at: https://www.mdpi.com/article/10.3390/cancers15143542/s1, Table S1: PRISMA Checklist; Table S2: Keywords and search results in different databases.
